# Beta-informativeness-diffusion multilayer graph embedding for brain network analysis

**DOI:** 10.3389/fnins.2024.1303741

**Published:** 2024-03-08

**Authors:** Yin Huang, Ying Li, Yuting Yuan, Xingyu Zhang, Wenjie Yan, Ting Li, Yan Niu, Mengzhou Xu, Ting Yan, Xiaowen Li, Dandan Li, Jie Xiang, Bin Wang, Tianyi Yan

**Affiliations:** ^1^College of Computer Science and Technology (College of Data Science), Taiyuan University of Technology, Taiyuan, China; ^2^School of Life Science, Beijing Institute of Technology, Beijing, China; ^3^School of Mechatronical Engineering, Beijing Institute of Technology, Beijing, China; ^4^Translational Medicine Research Center, Shanxi Medical University, Taiyuan, China; ^5^Computer Information Engineering Institute, Shanxi Technology and Business College, Taiyuan, China

**Keywords:** brain network, beta-informativeness-diffusion, graph embedding, schizophrenia, bipolar disorder

## Abstract

Brain network analysis provides essential insights into the diagnosis of brain disease. Integrating multiple neuroimaging modalities has been demonstrated to be more effective than using a single modality for brain network analysis. However, a majority of existing brain network analysis methods based on multiple modalities often overlook both complementary information and unique characteristics from various modalities. To tackle this issue, we propose the Beta-Informativeness-Diffusion Multilayer Graph Embedding (BID-MGE) method. The proposed method seamlessly integrates structural connectivity (SC) and functional connectivity (FC) to learn more comprehensive information for diagnosing neuropsychiatric disorders. Specifically, a novel beta distribution mapping function (beta mapping) is utilized to increase vital information and weaken insignificant connections. The refined information helps the diffusion process concentrate on crucial brain regions to capture more discriminative features. To maximize the preservation of the unique characteristics of each modality, we design an optimal scale multilayer brain network, the inter-layer connections of which depend on node informativeness. Then, a multilayer informativeness diffusion is proposed to capture complementary information and unique characteristics from various modalities and generate node representations by incorporating the features of each node with those of their connected nodes. Finally, the node representations are reconfigured using principal component analysis (PCA), and cosine distances are calculated with reference to multiple templates for statistical analysis and classification. We implement the proposed method for brain network analysis of neuropsychiatric disorders. The results indicate that our method effectively identifies crucial brain regions associated with diseases, providing valuable insights into the pathology of the disease, and surpasses other advanced methods in classification performance.

## 1 Introduction

The human brain represents an intricate network comprising interconnected regions in both structure and function (Cao et al., 2020). Anomalous wiring within the brain network may result in brain dysfunction (Van Den Heuvel et al., 2013). Neuropsychiatric disorders encompass a range of neurological diseases affecting the brain, characterized by cognitive dysfunction as a central symptom. Previous research has suggested that many neuropsychiatric disorders (such as schizophrenia, bipolar disorder, and Alzheimer’s disease) are caused by damage to the brain’s internal nervous system (Liu et al., 2018; Lian et al., 2020), leading to dysconnectivity between distinct brain regions (Yan et al., 2018; Wang et al., 2022). In medical physiology, neuroimaging techniques have rapidly evolved to provide critical insights into the diagnosis of neuropsychiatric disorders (Dubois and Adolphs, 2016; Cui et al., 2022; Liu et al., 2022a).

Brain networks derived from various neuroimaging modalities have been extensively used to analyze neuropsychiatric disorders. According to graph theory, a brain network comprises nodes and edges, with nodes denoting distinct brain regions, and edges signifying either physical connections or pairwise similarity. Diffusion tensor imaging (DTI) and functional magnetic resonance imaging (fMRI) are two frequently employed neuroimaging techniques. DTI reveals the physical connections between distinct brain regions, serving as a structural connectivity (SC) to build the structural brain network. fMRI captures the temporal correlation between blood-oxygen-level-dependent (BOLD) signals across various brain regions, which is normally treated as functional connectivity (FC) to establish the functional brain network (Osipowicz et al., 2016). Some methods relying on structural or functional brain networks have been effectively employed to identify potential biomarkers in the diagnosis of neuropsychiatric disorders. For example, Zhang et al. (2018) proposed ordinal patterns (e.g., subgraphs and motifs) containing weighted edge sequences for the connectivity analysis of brain networks. Huang et al. (2020a) employed SGNS to extract embedding features of structured brain networks and aligned these node representations through orthogonal transformations, then computed feature distances for brain disease diagnosis. Graph embedding methods, such as node2vec, are also widely used to extract node-level feature vectors of brain networks for brain disease analysis, which capture subtle structural changes in the brain network and contain richer information (Rahimiasl et al., 2021; Ramesh Kumar Lama and Kwon, 2021). These approaches are typically focused on either SC or FC, thereby only considering node interactions within a single modality. In practice, different modalities provide possibilities to analyze brain diseases from multiple perspectives (Dai et al., 2019; Zhang et al., 2021); integrating multiple modalities has been shown to be more effective than using a single modality in brain network analysis (Yan et al., 2020).

In recent years, A variety of approaches have emerged to combine SC and FC to perform brain network analysis (Huang et al., 2020b; Song et al., 2023). These methods typically can be divided into two categories. The first category involves a data fusion strategy, considering SC and FC as multi-modal data and combining their features by employing established machine learning techniques. For example, Gao et al. (2020) proposed a multi-kernel SVM to integrate multi-modal MRI by exploiting the subspace similarity of the decomposition components in each modality. Lei et al. (2020) combined low-order self-calibrated functional and structural brain networks to perform joint multitask learning for the early diagnosis of Alzheimer’s disease. Mill et al. (2021) used univariate and multivariate methods to fuse structural MRI and functional connectivity features for diagnosing patients with prescription opioid use disorder. These methods view SC and FC as separate modalities to extract latent node representations, neglecting the potential complementary information that exists between the modalities. The other category refers to a guiding strategy, which involves utilizing one modality to aid another in extracting features or leveraging multi-modal data to construct a unified brain network. For instance, Huang et al. (2020b) proposed an attention-diffusion-bilinear neural network for brain network analysis, in which node interactions in structural brain networks are used to further guide diffusion processes in functional brain networks to generate new node representations. Zhu et al. (2021) proposed a unified brain network construction framework, using a low-rank representation to build correlation models of all brain regions in functional data, simultaneously embedding local manifolds with structural data into the model to fuse multi-modal features. Liu et al. (2022b) utilized machine learning to extract important features from a structural graph network and exploited these features to adjust the corresponding edge weights in a functional graph network, which serves as an input to a multilayer GCN to achieve disease classification. However, these methods lead to each subject ultimately having only one brain network, thereby losing the unique characteristics of each modality’s brain network (Zhu et al., 2022). It has been proved that some internal properties within the brain network play a pivotal role in the analysis of brain networks (Wang et al., 2017; Yan et al., 2019). However, these multi-modal brain network analysis methods cannot adequately balance both the utilization of complementary information and the preservation of unique characteristics from various modalities.

To tackle this challenge, we propose a Beta-Informativeness-Diffusion Multilayer Graph Embedding (BID-MGE) method to learn holistic information for brain network analysis. Specifically, to maximize the preservation of each modality’s unique characteristics, we design a multilayer brain network, the functional layer of which is built through the guidance of its structural layer, and inter-layer connections are defined by node informativeness. Then, the multilayer informativeness diffusion first selects a more informative layer depending on node informativeness to exploit complementary information between modalities through wider node interactions. Within each layer, traversing nodes based on SC or FC capture the unique characteristics of each modality. Through propagating node features from a selected node to all its linked nodes in a diffusion manner, more comprehensive information is therefore considered in feature learning. In addition, beta mapping further assists the diffusion process to extract more discriminative features by refining crucial connectivity. Finally, to compare and analyze differences between different groups, we reconfigure node representations by PCA and then compute cosine distances with reference to multiple templates for statistical analysis and classifications. The statistical analysis is conducted on the node distances. For the classifications, the network distance serves as input into the Support Vector Machine (SVM) for identifying the label of each network.

The principal contributions of this study are as follows:

1. Beta mapping to refine the connectivity information of each modality. The refined information helps direct the diffusion process towards important brain region to capture discriminative features.
2. We proposed a novel framework for constructing a multilayer brain network, in which the inter-layer connections are based on node informativeness, and the network scale is optimized by the structural layer.
3. The multilayer informativeness diffusion learns complementary information and unique characteristics from various modalities. It is also an unsupervised embedding technique that only needs low time and space complexity and has no sample size limitations.
4. We validated the efficacy of our method on actual neuropsychiatric disorder datasets through two group-level analyses.

## 2 Proposed method

**Figure 1:**
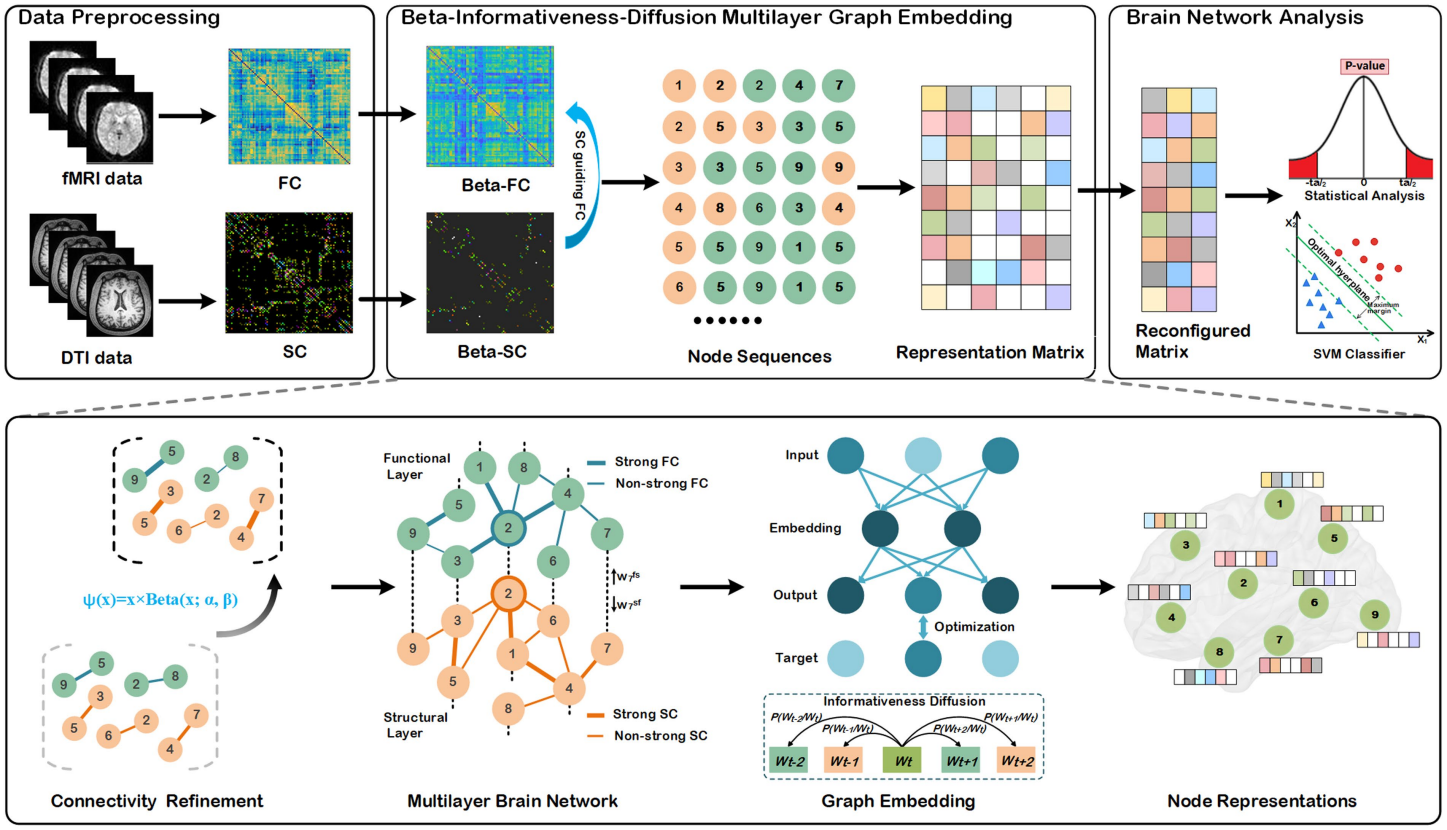
Architecture of the proposed BID-MGE method for brain network analysis. There are three modules in our method: a data preprocessing module, beta-informativeness-diffusion multilayer graph embedding module, and brain network analysis module. The data preprocessing module transforms the DTI and fMRI data into a structural and functional connectivity matrix. The Beta-Informativeness-Diffusion multilayer graph embedding module integrates SC and FC for generating node representations with comprehensive information of the brain network. The brain network analysis module consists of a statistical analysis and classifications.

The entire processes of our method are depicted in Figure 1, comprising three primary components: data preprocessing, node representation learning, statistical analysis, and disease classifications. We describe each component of the BID-MGE method in detail below.

### 2.1 Data preprocessing

Throughout the experiments, we utilized two types of data: MRI images and clinical scores. The MRI images encompass both DTI and resting-state fMRI (rs-fMRI), which require different preprocessing. The specific steps are described below.

DTI is preprocessed using PANDA toolboxes (Cui et al., 2013). First, the initial images go through head motion correction and eddy current distortion. Second, the fractional anisotropy (FA) is computed for every voxel, followed by registering the FA images in the original space to the T1-weighted images using an affine transformation. Third, we employ the Anatomical Automatic Labeling (AAL) atlas to delineate and mark the regions of interest (ROI) within the DTI data, and then reconstruct WM pathways (fibers or tracts) via a deterministic white matter tractography method (Mori and van Zijl, 2002). Finally, we acquire the count of fibers that connected any two brain regions from DTI data.

The rs-fMRI data is preprocessed using DPABI (Yan et al., 2016). Before starting the preprocessing, we discarded the initial 10 time points due to the incipient signal fluctuation. Subsequently, head motion and slice timing corrections are applied to each subject. Then, the T1 image is aligned with the central rs-fMRI image with corrected head movement. The functional images are resampled to 3-mm isotropic voxels and then subjected to spatial smoothing using a 4-mm full-width half-maximum (FWHM) Gaussian kernel. Several interfering signals, such as head motion signals, and cerebrospinal fluid are regressed from the image. Low-frequency drift and high-frequency noise are removed by linear detrending and bandpass filtering (0.01–0.25 Hz). Ultimately, the average time series are extracted from brain regions parcellated according to the AAL atlas.

### 2.2 Structural and functional brain network construction

Graphs provide a useful abstraction for representing many complex relationships in reality. In general, a weighted graph is denoted as G=(V,E,W) , where $V=\left\{v_{1},v_{2},\ldots ,v_{n}\right\}\;$ defines the set of the nodes, $E={\left\{e_{ij}\right\}}_{\left(i,j=1,2,\ldots \mathrm{,n}\right)}$ denotes the set of the edges, and W represents a connectivity matrix reflecting the strength of connectivity between any two nodes within the graph. Likewise, the human brain network can be abstractly denoted as such a graph. The graph’s nodes symbolize brain regions, while the edges represent the connections linking these regions. In our experiment, we adopt triples, $G_{s}=\left(V_{s},E_{s},W_{s}\right)\;$ and $G_{f}=(V_{f},E_{f},W_{f}$ ), to represent the structural and functional brain networks, respectively. Here, $V=V_{s}=V_{f}$ . Among them, $\;v^{s}\in V_{s}\;$ denotes a brain region in the structural brain network. $W_{s}$ signifies a structural connectivity matrix, with the weight $w_{ij}^{s}\in W_{s}$ for each $e_{ij}^{s}\in E_{s}$ calculated by the count of fibers divided by the sum of two interconnected surface areas of ROIs. $\;v^{f}\in V_{f}$ represents a brain region within the functional brain network $,W_{f}\;$ refers to a functional connectivity matrix, with the weight $w_{ij}^{f}\in W_{f}$ for each $e_{ij}^{f}\in E_{f}$ determined by computing the Pearson correlation among the average time series of the brain regions. Notably, as the negative correlation coefficients have no clear biological explanations, it is common practice to set these negative values to zero (Murphy et al., 2009; Cao et al., 2020). Additionally, the self-correlations coefficients are also set to zero (Rubinov and Sporns, 2010).

### 2.3 Connectivity information refinement

To extract more discriminative features, the following mapping function (beta mapping) as shown in Eq. 1, has been proposed to refine the connectivity information of the brain.


(1) ψ(x)=x×Beta(x;α,β).

**Figure 2:**
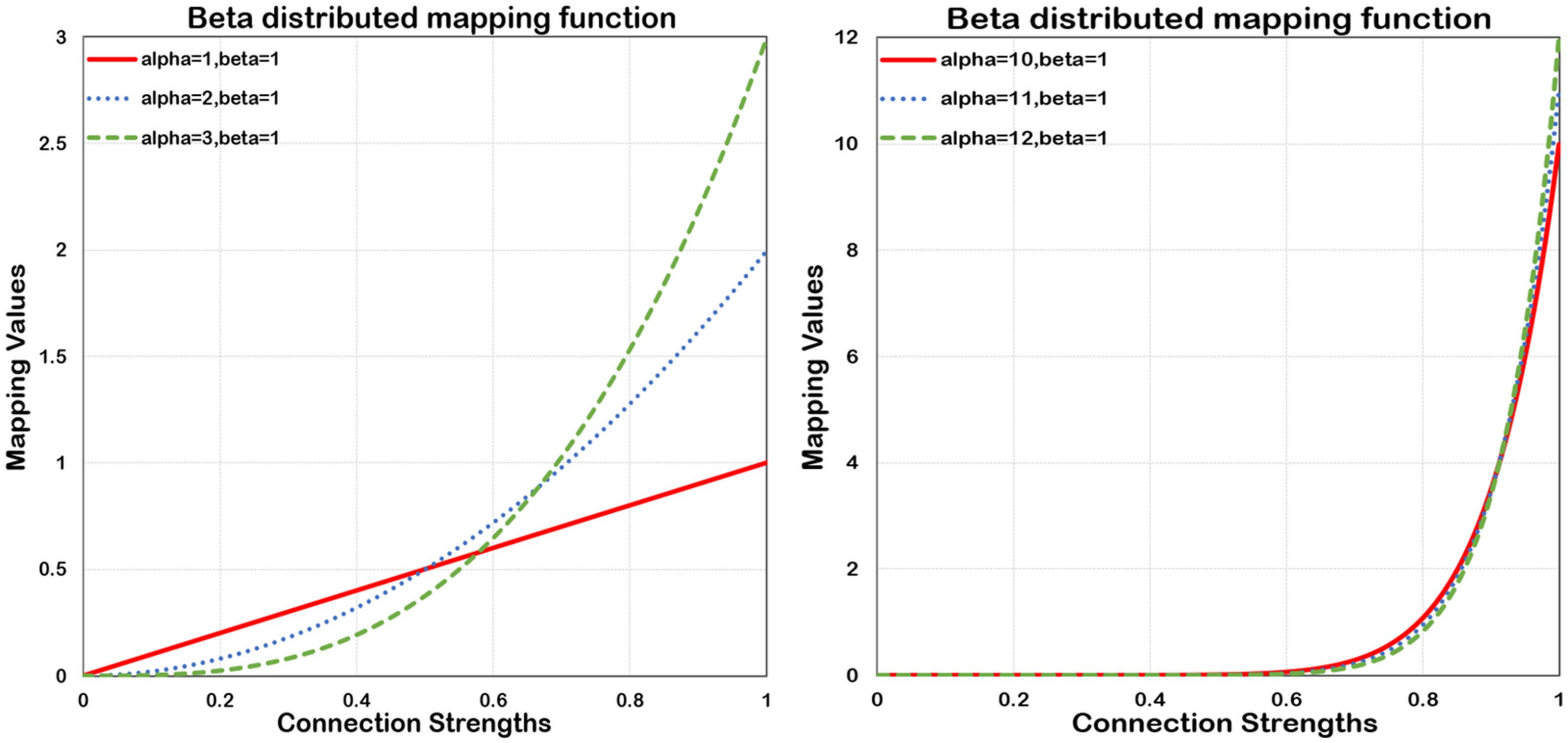
Beta distributed mapping function. Beta mapping with different values of α and a fixed *β* = 1. As *α* increases, the squeezing and expanding properties become stronger.

Where Beta is a continuous probability distribution function on the range [0,1]. The parameters α and β, both more than zero, determine the shape of its distribution. The shape can be concave, convex, monotonically increasing, monotonically decreasing, and curved or straight. However, the probability density function (PDF) of Beta is monotonically ascending only in the case of α ≥ 1 and β ≤ 1, which maps smaller values to nearly zero numbers, and larger values to more significant numbers, thereby allowing for its compression and expansion properties. The Beta’s compression and expansion properties enable ψ(x) to scale the input values. Considering two typical values of connection strength, 0.5 and 0.9, the value 0.5 normally happens between nodes. In contrast, the value 0.9 rarely occurs, and it also implies a strong connection between connected nodes. Without using beta mapping, the latter value is merely 80% stronger than the former. However, by employing beta mapping with *α* =2 with *β* = 2, the latter transforms to 1.62, signifying a 224% increase in strength. In Figure 2, we present the beta mapping ψ(x) for different values of α with β constant 1. The larger *α* means more significant compression and expansion properties. The maximum value of ψ(*x*) is equal to α when x = 1. ψ(*x*) makes it possible to refine the essential connections and eliminate negligible information. Eventually, the connectivity matrices $W_{s}$ and $W_{f}$ are converted to $BW_{s}$ and $BW_{f}$ , respectively.

### 2.4 Structure-guided multilayer brain network construction

A multilayer brain network comprises two layers: a structural layer and a functional layer that correspond to the structural and functional brain networks, respectively. For the structural layer, its edges are determined from the structural connectivity matrix $BW_{s}$ . Therefore, this layer is inherently a sparse network, and the number of edges is also fixed. For the functional layer, the edges are derived from the functional connectivity matrix $BW_{f}$ , which is almost fully connected, and some of the connections are negligible, which also increases the computation time of the multilayer brain network, so only some of the important connections in $BW_{f}\;$ will be used to build the functional layer instead of all of them. In this study, we adopt the structural layer to guide the selection of edges for building the functional layer, determining its network scale so that it is comparable in scale to the structural layer. Specifically, we first calculate the average edge number of all nodes within the structural layer, denoted by $avg_{s}$ . If the given network is undirected, $avg_{s}=2\times \left|E_{s}\right|/n$ , otherwise, $avg_{s}=\left|E_{s}\right|/n$ . Then, for each node $v^{f}$ , the top $\theta \times avg_{s}\;$ edges are selected to construct the functional layer in terms of the connection values in $BW_{f}$ , where θ is the network scale parameter, $\theta \in {\mathbb{R}}_{+}$ . Finally, inter-layer edges (directed and weighted) are used to connect the corresponding nodes in the structural and functional layers to constitute a multilayer brain network. The weights of these edges depend on node informativeness. The notion of node informativeness will be explained later.

### 2.5 Multilayer informativeness diffusion

We propose a graph embedding technique based on multilayer informativeness diffusion, which learns node representations by intelligently traversing the nodes between structural and functional layers in a diffusion manner. Whenever the diffusion process reaches a node, our goal is to select a more informative layer by assessing the informativeness of the current node in its corresponding layer.

A node that has strong connections to many nodes is less similar to its neighbors, while a node strongly connected to only a few nodes is more similar to its neighbors. The latter node also means more informativeness (Ribeiro et al., 2017). For the diffusion process, it is crucial to traverse nodes that have more informativeness. In this study, we suppose that a strong connection refers to an edge with a weight exceeding the average weight of its network layer. Consequently, we define $T_{i}^{s}$ as the collection of neighbors’ non-strong connection with node $v_{i}^{s}$ in the structural layer, denoted as Eq. 2.


(2) $${Τ}_{i}^{s}=\{\;\upsilon_{j}^{s}\in V_{s}\;|w_{ij}^{s}\leq \frac{1}{\left|E_{s}\right|}\underset{e^{\prime }\in E_{s}}{\sum }w_{e^{\prime }}^{s}\}.\;\;$$

Each node in $T_{i}^{s}$ has an edge connected to $v_{i}^{s}$ with a weight not exceeding the mean weight of the structural layer. $\left|T_{i}^{s}\right|$ denotes the count of nodes that belong to $T_{i}^{s}$ . Similarly, $T_{i}^{f}$ for the functional layer is defined as Eq. 3:


(3) $${Τ}_{i}^{f}=\{\;\upsilon_{j}^{f}\in V_{f}\;|w_{ij}^{f}\leq \frac{1}{\left|E_{f}\right|}\underset{e^{\prime }\in E_{f}}{\sum }w_{e^{\prime }}^{f}\}.$$

Given the sets $T_{i}^{s}$ and $T_{i}^{f}$ , the informativeness of nodes $v_{i}^{s}$ and $v_{i}^{f}$ is defined as Eq. 4.


(4) $${Ι}_{i}^{s}=\ln\left(e+|{Τ}_{i}^{s}|\right),{Ι}_{i}^{f}=\ln\left(e+|{Τ}_{i}^{f}|\right).$$

Now, let us consider the inter-layer directed weighted edges. The weight is set as ${Ι}_{i}^{s}$ from the functional layer to the structural layer, and vice versa as ${Ι}_{i}^{f}$ . The diffusion process starts with selecting the structural or functional layer according to the weights of inter-layer directed edges. If the value of ${Ι}_{i}^{s}$ is high, the diffusion process will step into the structural layer. Otherwise, the functional layer will be chosen. We aim to step into a layer where the node possesses greater informativeness.

Subsequently, we formulate the probabilities of inter-layer and intra-layer diffusion for multilayer informativeness diffusion. Given a node $v_{i}$ , the probability of inter-layer diffusion is defined as Eq. 5:


(5) $$P\left(v_{i}^{s}|v_{i}^{f}\right)=\frac{{Ι}_{i}^{s}}{{Ι}_{i}^{s}+{Ι}_{i}^{f}},P\left(v_{i}^{f}|v_{i}^{s}\right)=\frac{{Ι}_{i}^{f}}{{Ι}_{i}^{s}+{Ι}_{i}^{f}}.\;\;$$

Where the likelihood of moving to a structural layer is represented as $P\left(v_{i}^{s}|v_{i}^{f}\right)$ , and vice versa for $P\left(v_{i}^{f}|v_{i}^{s}\right)$ . The probability of intra-layer diffusion delineates the likelihood of transitioning from the present vertex to the subsequent vertex within the layer. Suppose the diffusion process visited node $v_{k-1}^{l_{i}}$ at time t–1 and propagated to node $v_{k}^{l_{j}}$ at current time t, where $l_{i}$ and $l_{j}$ denote the corresponding layers $l_{i},l_{j}\in \left\{s,f\right\}$ . If the diffusion process steps into another layer at time t (i.e., $l_{i}\neq l_{j}$ ), $\;e_{\left(k-1,k\right)}^{l_{j}}\notin E_{l_{j}}$ , otherwise (i.e., $l_{i}=l_{j}$ ), $e_{\left(k-1,k\right)}^{l_{j}}\in E_{l_{j}}$ . For $\;e_{\left(k-1,k\right)}^{l_{j}}\notin E_{l_{j}}$ , The selection probability of the next node depends entirely on the weight of the edges connecting to $v_{k}^{l_{j}}$ in layer $l_{j}$ . In other cases, the intra-layer diffusion probabilities follow the unnormalized transition probabilities in node2vec (Grover and Leskovec, 2016). Hence, we define the probability of intra-layer diffusion (i.e., the probability of selecting the next node $v_{k+1}^{l_{j}}$ in layer $l_{j}\;$ at time *t* + 1) as Eq. 6:


(6) $$\begin{array}{l} P\left(v_{k+1}^{l_{j}}|v_{k}^{l_{j}},v_{k-1}^{l_{i}}\right)\\ =\{\begin{array}{c} w_{\left(k,k+1\right)}^{l_{j}},if\;e_{\left(k-1,k\right)}^{l_{j}}\notin E_{l_{j}}\\ \frac{1}{p}w_{\left(k,k+1\right)}^{l_{j}},if\;e_{\left(k-1,k\right)}^{l_{j}}\in E_{l_{j}}\wedge d_{\left(k-1,k+1\right)}^{l_{j}}=0\\ w_{\left(k,k+1\right)}^{l_{j}},if\;e_{\left(k-1,k\right)}^{l_{j}}\in E_{l_{j}}\wedge d_{\left(k-1,k+1\right)}^{l_{j}}=1\;\\ \frac{1}{q}w_{\left(k,k+1\right)}^{l_{j}},if\;e_{\left(k-1,k\right)}^{l_{j}}\in E_{l_{j}}\wedge d_{\left(k-1,k+1\right)}^{l_{j}}=2 \end{array}. \end{array}$$

Here, $\;d_{\left(k-1,k+1\right)}^{l_{j}}$ represents the unweighted path length between two nodes, $v_{k-1}^{l_{j}}$ and $v_{k+1}^{l_{j}}$ . For parameters p and q, both are greater than 0. Parameter p determines the probability of traversing the recently visited node $v_{k-1}^{l_{i}}$ , and parameter q controls the search to proceed in either a BFS or DFS manner. If *q* > 1, the diffusion process prefers nodes closer to node $v_{k-1}^{l_{i}}$ . If *q* < 1, the diffusion process tends to visit nodes farther away from it.

The multilayer informativeness diffusion is performed as follows: at a given time point of the diffusion process, a node is on either the structural or functional layer. The diffusion process first evaluates the informativeness of the node in each layer to determine which layer to enter next, then traverses the node according to the transition probabilities. The selected node is added to node sequences after discarding its layer information, which ensures each node corresponds to only one node representation. We repeatedly perform the above steps *λ* times, where *λ* signifies the truncated walk length starting from a node.

After generating the necessary number of node sequences for every node, learning node representation is achieved using the following objective function (Eq. 7), optimizing the log-probability of a node observing its context within the node sequence, given by F :


(7) $$\underset{F}{\max}\underset{v\in V}{\sum }\log P(N\left(v\right)|F\left(v\right))=\underset{F}{\max}\underset{v\in V}{\sum }\underset{u\in N\left(v\right)}{\sum }\log P(u|F\left(v\right)).$$

Let $F:V\to {\mathbb{R}}^{d}$ be a learnable projection function mapping nodes to vector representations. Here, parameter d fixes the dimensions of the node representation. Correspondingly, F specifies a parameter matrix of size n×d , representing the node representation. N(v) is the neighborhood of node v in a diffusion process. To render the optimization problem tractable, we also apply two criterion assumptions: conditional independence and feature space symmetry (Grover and Leskovec, 2016). The above optimization function is simplified (Eq. 8):


(8) $$\underset{F}{\max}\underset{v\in V}{\sum }\underset{u\in N\left(v\right)}{\sum }\left(-\log Z_{v}+F\left(u\right).F\left(v\right)\right).$$

The partition function $\;Z_{v}=\underset{v^{\prime }\in V}{\sum }\exp\left(F\left(v^{\prime }\right).F\left(v\right)\right)$ can be estimated using negative sampling. The model parameters denoting the feature F in Eq. 8 can be optimized through stochastic gradient ascent.

### 2.6 Node representation reconfiguration

A particular dimension within a node representation may encompass varying latent concepts across different networks. Hence, these representations have to be reconfigured sequentially to ascertain the importance of individual features (Salsabilian and Najafizadeh, 2020). To accomplish this objective, we adopt PCA, which also serves as information compression. We retain top k principal components ( k<d ) and transform the representation matrix $F^{n\times d}$ into a reconfigured representation matrix $A^{n\times k}$ in an important sequential manner $\;\left(p_{1},p_{2},\ldots .p_{k}\right)$ , where $p_{i}$ represents the i th principal component as a column vector and the row j of A , $\;A_{j}$ , denotes the j th reconfigured node representation.

### 2.7 Cosine distance computation

Given two vector representations, $A=\left(x_{1,}x_{1,}\ldots ,x_{t}\right)$ and $\;B=\left(y_{1,}y_{1,}\ldots ,y_{t}\right)$ , the cosine distance between A and B can be calculated as Eq. 9:


(9) $$\mathrm{Cos}Dist\left(A,B\right)=1-\cos\left(A,B\right)=\frac{A_{2}B_{2}-A\cdot B}{{\mathrm{||A||}}_{2}{\mathrm{||B||}}_{2}}.$$

which reflects the differences between vector representations. The smaller the distance is, the more similar the vector representations are. Nevertheless, because of lacking shared reference coordinates, such pairwise distances are not directly employed in the group-level analysis (Huang et al., 2020a). To compare differences between different groups, we propose node distance and network distance, with reference to common coordinates at the node-level and network-level, respectively.

#### 2.7.1 Node distance

After reconfiguring node representations, we calculate the node distance. This node distance becomes smaller if nodes i and j are more similar in structure or function. First, we construct the reference template ${\left\{\tau_{1},\tau_{1},\cdots \tau_{n}\right\}}^{T}$ , where $\tau_{i}\in {\mathbb{R}}^{k}$ is the centroid node representation ( $\tau_{i}=\frac{1}{m^{c}}\overset{m^{c}}{\underset{r=1}{\sum }}A_{i}^{r}$ , where $m^{c}$ is the count of subjects with the same labeling). Second, we calculate the distances between nodes in the target network and those in the template. Given a target network $\;G_{t}\;$ and the reference template ${\left\{\tau_{1},\tau_{1},\cdots \tau_{n}\right\}}^{T}$ , a node distance vector $l=\left\{\iota_{1},\iota_{2},\cdots \iota_{n}\right\}$ can be obtained, here $\iota_{i}$ is the node distance between nodes $v_{i}$ in both networks (i.e., $\iota_{i}=CosDist\left(A_{i}^{t},\tau_{i}\right)$ ). Notably, the template can be designated as the HC template ${\left\{\tau_{1}^{h},\tau_{2}^{h},\cdots \tau_{n}^{h}\right\}}^{T}$ , the SZ template ${\left\{\tau_{1}^{s},\tau_{2}^{s},\cdots \tau_{n}^{s}\right\}}^{T}$ , and the BD template ${\left\{\tau_{1}^{b},\tau_{2}^{b},\cdots \tau_{n}^{b}\right\}}^{T}$ . Third, we utilize the node distance vector, generated for each subject, to compose a node distance matrix, $L^{m\times n}={\left\{l_{1},l_{2},\cdots l_{m}\right\}}^{T}$ , where $m=m^{h}+m^{s}+m^{b}$ and $m^{h}$ , $m^{s}$ , and $m^{b}$ are the number of HC, SZ and BD subjects, respectively. Each column, L[i] , can be subdivided into three parts based on the label of each network: $L^{h}\left[i\right],L^{s}\left[i\right]$ , and $L^{b}\left[i\right]$ . Using these node distances, the two-tailed *t*-test will be employed to recognize brain regions exhibiting structural or functional differences.

#### 2.7.2 Network distance

Moreover, the network distance can also be computed using reconfigured representations. First, node representations, ${Α}^{n\times k}$ , are concatenated to generate a network representation ${Α}^{\prime 1\times \left(n\times k\right)}$ for each network. To find the all-round network-level differences between groups, we construct the positive template $C^{+}=\left\{c_{1}^{+},c_{2}^{+},\cdots c_{n\times k}^{+}\right\}$ and the negative template $C^{-}=\left\{c_{1}^{-},c_{2}^{-},\cdots c_{n\times k}^{-}\right\}$ (i.e., $\;C^{+}=\frac{1}{m^{+}}\overset{m^{+}}{\underset{i=1}{\sum }}A_{i}^{\prime }$ , $C^{-}=\frac{1}{m^{-}}\overset{m^{-}}{\underset{i=1}{\sum }}A_{i}^{\prime }$ , where $m^{+}$ , $m^{-}$ is the respective count of positive and negative samples). According to these templates, a network distance matrix $H\in {\mathbb{R}}^{\left(m^{+}+m^{-}\right)\times 2}$ is proposed to depict the network distance between each network and reference templates. For instance, the network distance between the target network $\;G_{a}\;$ and two templates can be computed as $H_{\left(a,1\right)}=CosDist\left(A_{a}^{\prime },C^{+}\right),H_{\left(a,2\right)}=CosDist\left(A_{a}^{\prime },C^{-}\right)$ . H reflects the network distance between each network and the corresponding positive and negative templates, with the first and second columns of H representing the two kinds of distances.

### 2.8 Statistical analysis and classification

This study performs *t*-tests on each column L[i] to identify significantly different brain regions, considering different templates as the references. The Bonferroni correction (Bonferroni *p* < 0.05) is employed to address the issue of node-level multiple comparisons. For disease classification, the network distance matrix, H , serves as the input for the SVM classifier to determine the corresponding labels.

## 3 Experiments

### 3.1 Dataset

The proposed method is evaluated using the Consortium for Neuropsychiatric Phenomics (CNP) database (Poldrack et al., 2016), which is hosted on OpenfMRI (www.openfmri.org). In addition, the CNP dataset also contained substantial demographic information, neuropsychological assessments, and neurocognitive task results. The study collected 147 subjects with DTI and rs-fMRI brain imaging data, including 50 healthy controls (HC), 48 SZ patients, and 49 BD patients. All participants were between 21 and 50 years of age. A two-tailed *t*-test was performed for age and sex, both of which were not significantly different. Table 1 presents detailed demographic information about the subjects. All brain imaging data were acquired using a Siemens Trio scanner. The parameters for obtaining DTI data were as follows: slices = 176, slice thickness = 1 mm, T*R* = 1,900 ms, echo TE = 2.26 ms, FOV = 250 mm, flip angle = 90°, and the acquisition matrix = 256 × 256. The parameters of collecting rs-fMRI data were as follows: slices = 34, slice thickness = 4 mm, T*R* = 2,000 ms, TE = 30 ms, FOV = 192 mm; flip angle = 90°, and the acquisition matrix = 64 × 64.

**Table 1:** The detailed demographic information of participants used in this study.

Name	Number	Age (mean ± std)	Gender (female / male)
Healthy controls (HC)	50	32.9 ± 8.2	20 / 30
Schizophrenia (SZ)	48	35.8 ± 8.7	13 / 35
Bipolar disorder (BD)	49	35.3 ± 8.9	21 / 28

### 3.2 Node distance analysis

**Figure 3:**
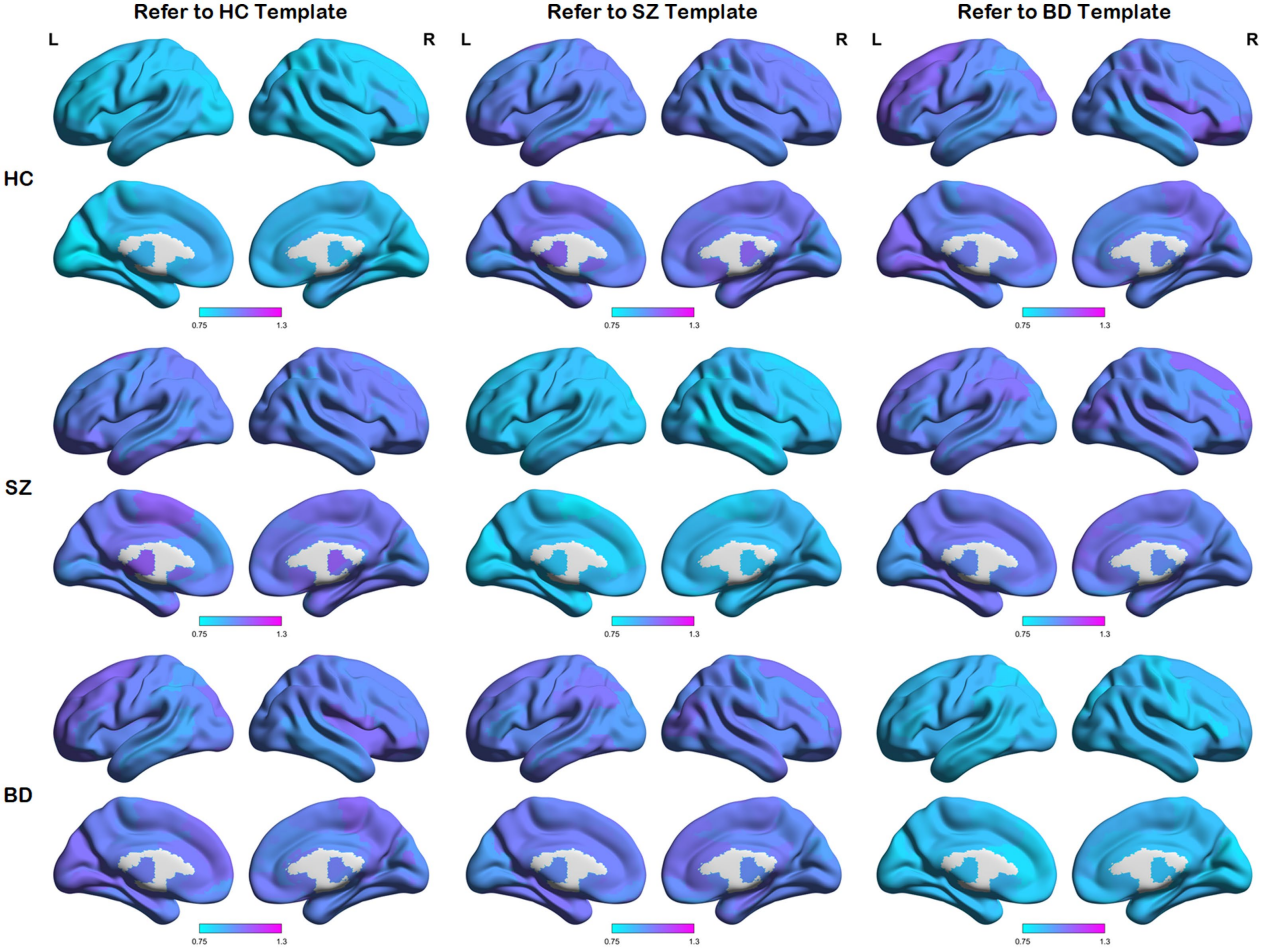
Maps of average node distances. Average node distances between each group and three templates (i.e., the HC template, SZ template, and BD template).

We first calculated node distances between each network and the reference templates (i.e., the HC template, SZ template, and BD template). These average node distances for each group (i.e., the HC group, SZ group, and BD group) are presented in Figure 3. A larger node distance means greater individual differences in that brain region. Node distances between each group and their homologous templates are consistently small, as shown in the main diagonal line of Figure 3. Some regions of the brain exhibit larger node distances between each group and their heterogeneous templates. In addition, along the main diagonal line, node distances show a similar distribution in symmetrical positions. For example, HC subjects refer to the SZ template and SZ patients refer to the HC template, as the node distance reflects the same node differences from opposite perspectives. These detailed node differences are revealed through the following statistical analysis.

**Figure 4:**
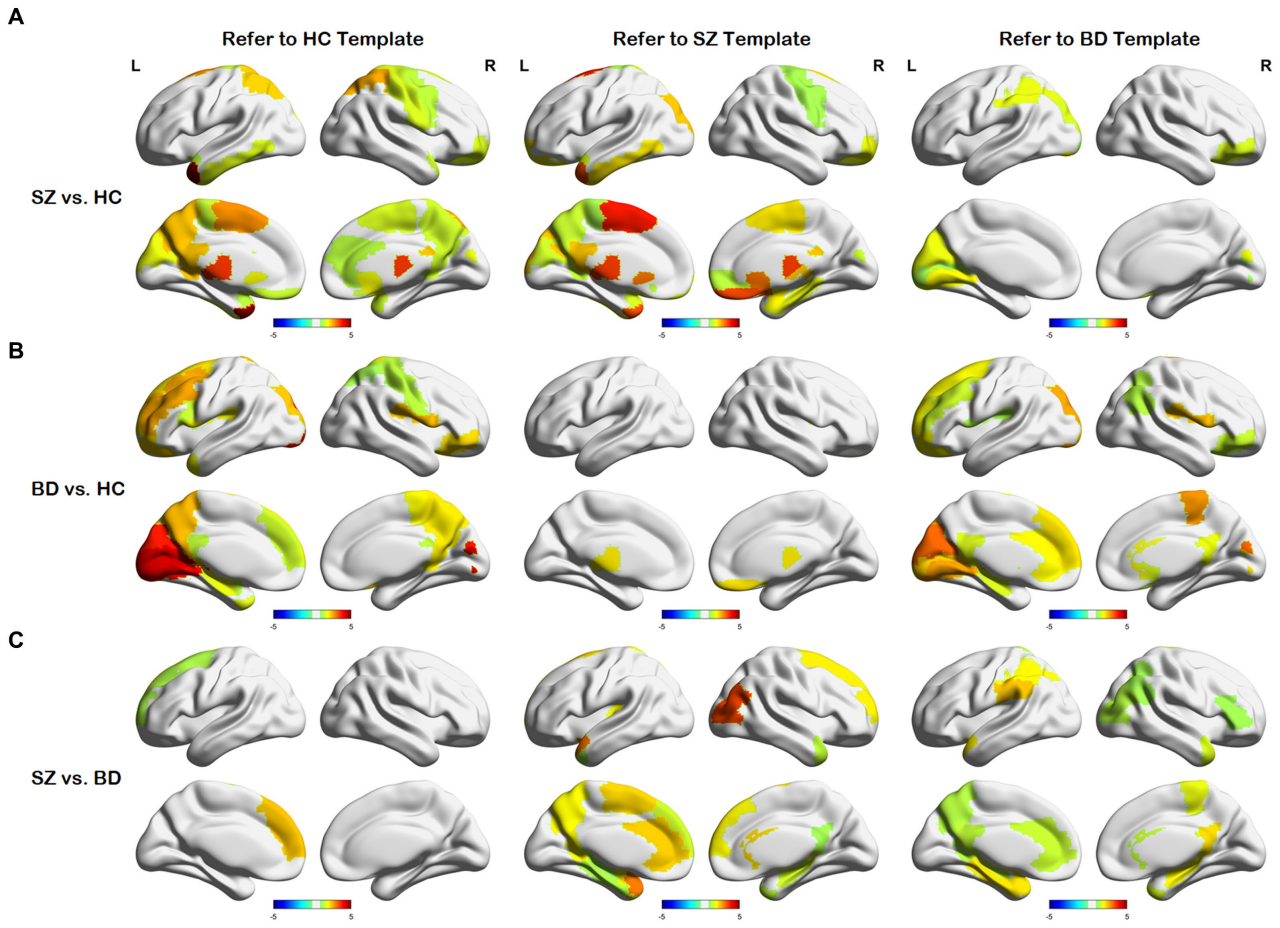
Differences in node distances between different groups with reference to the three templates. **(A)** Node differences between the SZ and HC groups. **(B)** Node differences between the BD and HC groups. **(C)** Node differences between the SZ and BD groups.

After obtaining the node distance matrix L , we performed the statistical test on each column of L (i.e., $L^{h}\left[i\right]$ , $L^{s}\left[i\right]$ , and $L^{b}\left[i\right]$ ). The nodes with significant differences between any two of the HC, SZ, and BD groups are presented in Figure 4. We discovered that only a few nodes are significantly different on their common heterogenous templates for two groups, as shown in the sub-diagonal line in Figure 4. Most of the nodes with significant differences are concentrated on any homologous template for two groups. As shown in Figure 4A, nodes with differences between SZ and HC groups are concentrated in the thalamus, gyrus rectus, precuneus, posterior cingulate gyrus, middle frontal gyrus orbital and motor area. From Figure 4B, these nodes exhibiting differences between BD and HC groups primarily localize in the frontal lobe, cuneus, lingual gyrus, rolandic operculum, and hippocampus. Figure 4C shows nodes with differences between the SZ and BD groups are mainly the posterior cingulate gyrus, parahippocampal gyrus, precuneus, and hippocampus. Additionally, we observed that brain regions with significant differences in the homologous templates related to both groups are not completely consistent. For example, the superior parietal gyrus and postcentral gyrus only show differences on the HC template, whereas the amygdala and parahippocampal gyrus orbital only present differences on the SZ template. This might be attributed to the following factors: (1) The diverse causes of different neuropsychiatric disorders and (2) the inherent large distances between templates.

### 3.3 Network distance visualization

**Figure 5:**
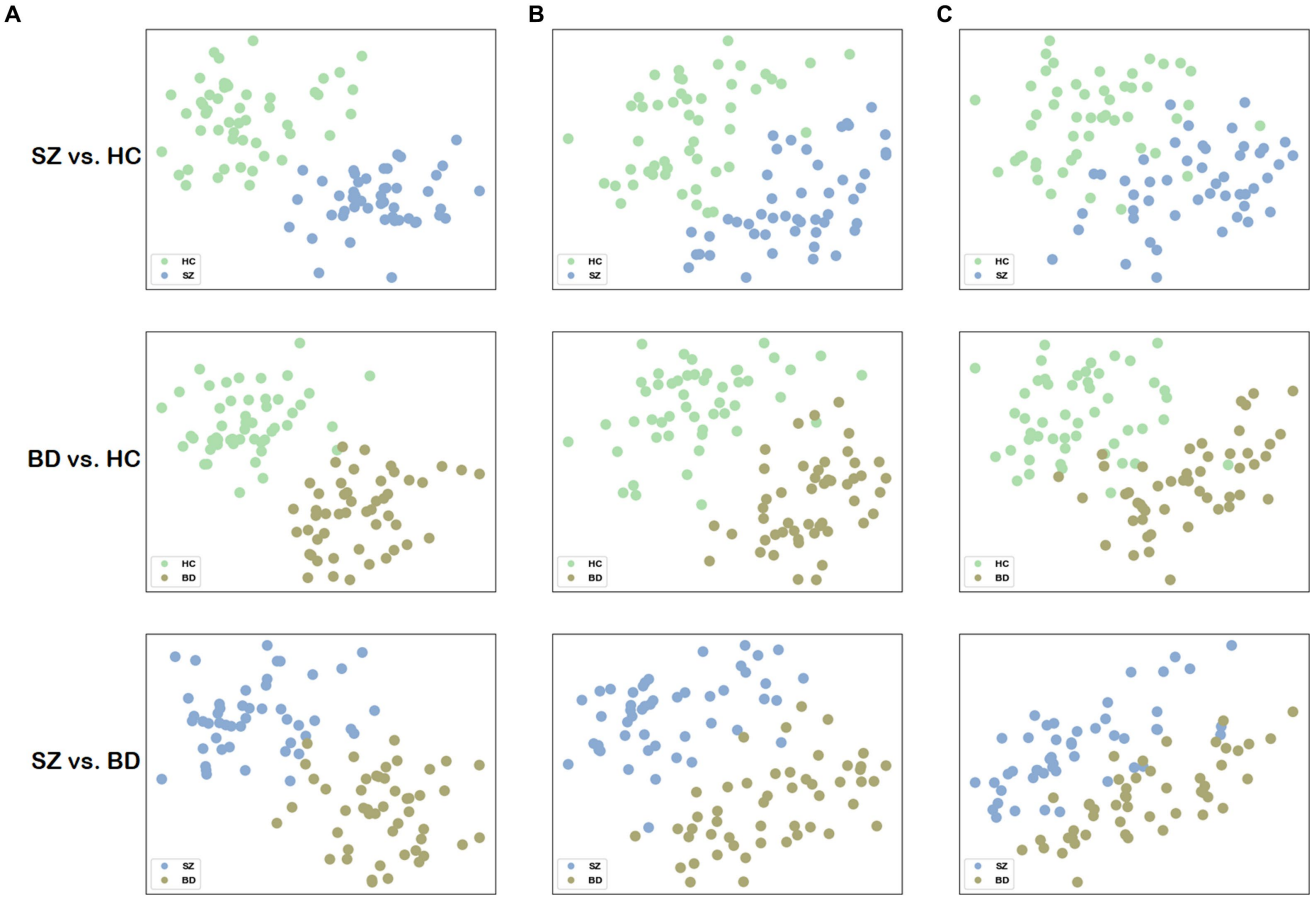
Visualization of the network distance matrix. **(A)** Scatter plots of the network distance matrix for our method. **(B)** Scatter plots of the network distance matrix for the structural brain network. **(C)** Scatter plots of the network distance matrix for the functional brain network.

To visualize the network distance, we mapped the distance matrix H onto a two-dimensional plane, where the first and second columns of H are assigned to the horizontal and vertical axes, respectively. To facilitate comparison, we also visualized the network distance for structural and functional brain networks, the node representations of which are extracted by node2vec, and the parameter settings are the same as our method. The merit of network distance is estimated by observing how clustered the points belonging to the same class are. Figure 5 visualizes the 2D scatter plots of these distance matrices in three classification combinations. The distance matrix generated by building a multilayer brain network with our approach outperforms using single-modal brain networks. Consequently, based on this distance matrix H , distinct groups can be easily distinguished by employing some machine learning methods (e.g., SVM).

### 3.4 Performance evaluation

For the evaluation of classification performance, we employed classification accuracy (ACC), sensitivity (SEN), specificity (SPE), and the area under the receiver operating characteristic (ROC) curve (AUC). These metrics are defined as Eqs. 10-12:


(10) $$ACC=\frac{TP+TN}{TP+FN+TN+FP}$$

(11) $$SEN=\frac{TP}{TP+FN}\;$$

(12) $$SPE=\frac{TN}{TN+FP}.$$

where TP, TN, FP, and FN denote the number of true positives, true negatives, false positives, and false negatives, respectively.

### 3.5 Classification performance

To evaluate the efficacy of our method in distinguishing patients from healthy controls (i.e., SZ vs. HC and BD vs. HC), we conducted a comparison with several baseline methods. The baseline models include state-of-the-art brain network analysis methods.

SVM (Atlas-based) (Tripathi et al., 2017): uses an atlas-based segmentation method to extract multiple known disease-related regions of interest and then employs gray-matter voxel-based intensity variations and structural changes extracted with a spherical harmonic framework to learn the discriminative features.

H-FCN (Lian et al., 2020): proposes a hierarchical full convolutional network to automatically identify discriminative local plaques and regions, then jointly learns and fuses multi-scale feature representations to construct hierarchical classification models for AD diagnosis.

nSEAL (Huang et al., 2020a): defines a node-level structural embedding and alignment representation to accurately characterize the node-level structural information, and calculates distances at different scales based on the embedding representation for brain disease analysis.

DCNs (Jie et al., 2018): uses manifold regularized multi-task feature learning and multi-kernel learning to integrate both temporal and spatial variabilities of DCNs for brain disease diagnosis.

N2EN (Zhu et al., 2018): proposes a non-negative elastic-net based method to extract changes in brain functional connectivity. Then, a kernel discriminant analysis (KDA) is utilized to classify subjects with the selected discriminative brain connectivity features.

SVM (Multi-kernel) (Shao et al., 2020): uses a group-sparsity regularizer with a hypergraph-based regularization term to jointly select the common features of multiple modalities. Then, a multi-kernel SVM is utilized to integrate the features selected from different modalities for final classification.

3D-CNN (Masoudi et al., 2021): proposes a multimodal hierarchical fusion method based on attention mechanisms, selectively extracting features from MRI and PET while suppressing irrelevant information.

HebrainGNN (Shi et al., 2022): models the brain network as a heterogeneous graph with multiple types of nodes and edges. Then, a self-supervised pre-training strategy based on the heterogeneous brain network is proposed to solve the potential overfitting problem.

MME-GCN (Liu et al., 2022b): adopts XGBoost to extract important features from the structural brain network. These features are used to adjust the corresponding edge weights in the functional brain network. Finally, a multi-layer GCN is trained and applied to binary classification tasks.

OLFG (Chen et al., 2023): projects multiple modalities into a common latent space by orthogonal constrained projection with learning graph regularization terms to capture discriminative information, and adaptively ranks feature importance using a feature weighting matrix. Finally, the representations in the latent space are mapped to the target space for AD diagnosis.

Based on the inputs, we categorized these methods into two classes. One category only employs single-modal data as input, while the other incorporates multi-modal data. For a fair comparison, we either precisely reproduced these methods as mentioned in the article or utilized the code provided by the authors. In addition, all methods used identical training and test sets. The 10-fold cross-validation is employed to assess classification performance, repeating 10 times to derive the average performance.

**Figure 6:**
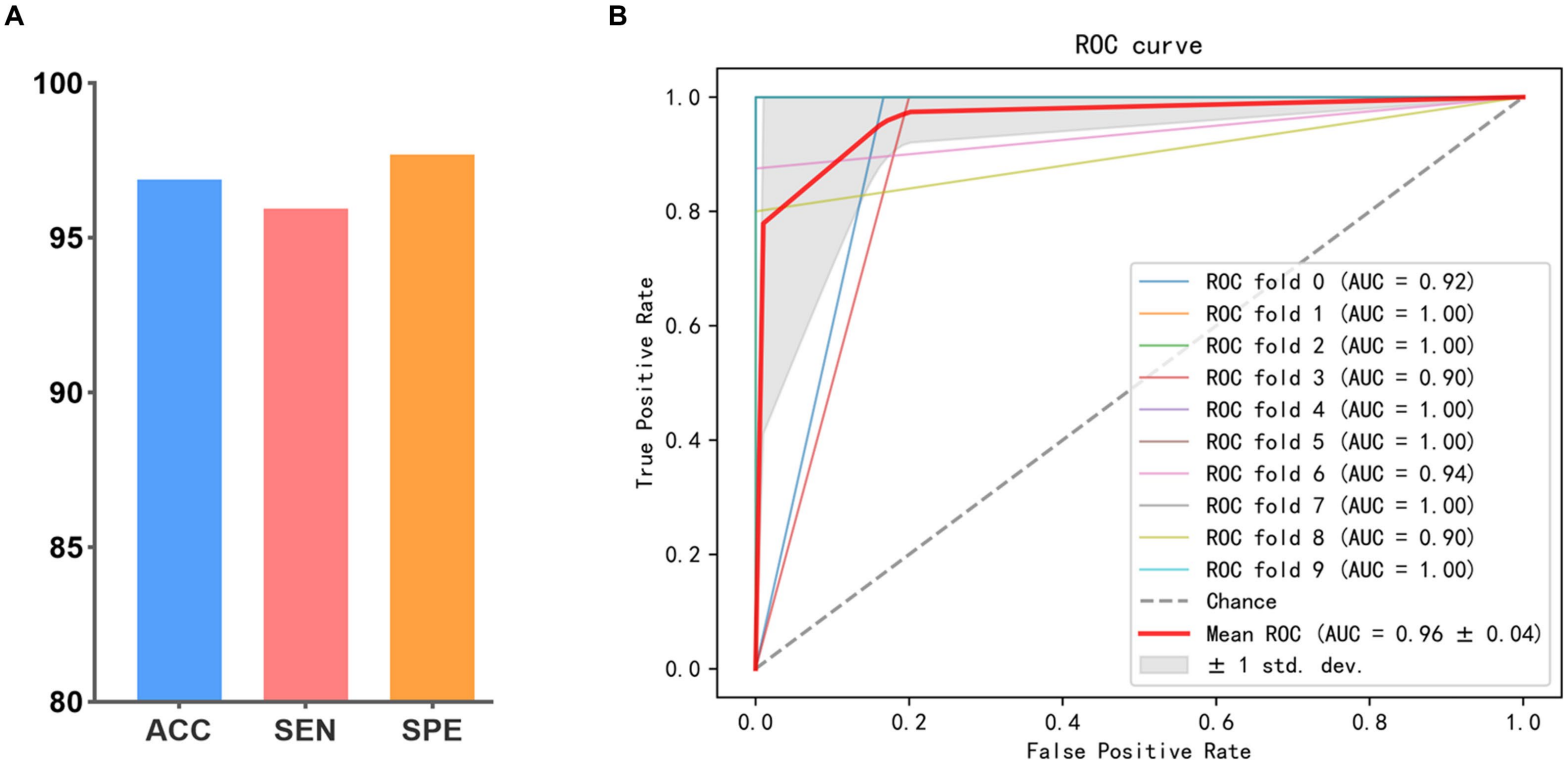
Classification performance in SZ vs. BD. **(A)** ACC, SEN, and SPE. **(B)** ROC curve.

The results of all methods are presented in Table 2. The accuracy values obtained from the proposed method in SZ vs. HC and BD vs. HC classification tasks achieve 99.07 and 98.80% respectively, which consistently outperforms all methods compared. Most multi-modal methods incorporating DTI and fMRI exhibit superior performance to single-modal methods using the DTI or fMRI. The accuracy of the majority of single-modal methods is below 95%, whereas multi-modal methods achieve an accuracy exceeding 95%. This verifies that combining SC and FC can offer complementary information, thereby enhancing the classification performance. Moreover, among all multi-modal methods, SVM (Multi-kernel) yields the lowest accuracy at 95.60 and 95.82%. The proposed BID-MGE method attains optimal performance on most evaluation metrics, surpassing the highest comparison method (OLFG) by approximately 2.00%. In addition, we observed that employing the embedding features directly as inputs to SVM for classification has a lower performance than some multi-modal brain network analysis methods (e.g., MME-GCN, 3D-CNN, and OLFG). This discrepancy arises from the substantial feature dimensionality resulting from concatenating all nodes, which is prone to causing a “dimensional disaster” and negatively impacting classification performance. Neural network methods, however, are better equipped to handle high-dimensional features. To further examine the sensitivity of the BID-MGE method for diverse neuropsychiatric disorders, we conducted a binary classification between SZ and BD. As shown in Figures 6A,B, our method also achieves a promising result with an ACC of 96.88, SEN of 95.94%, SPE of 97.11%, and AUC of 0.9682, which exceeds the latest neuroimaging and brain network research (Chen et al., 2017; Du et al., 2020).

**Table 2:** Performance of all comparative methods in SZ vs. HC and BD vs. HC classification.

Method	Modality	SZ vs. HC				BD vs. HC			
		ACC (%)	SEN (%)	SPE (%)	AUC	ACC (%)	SEN (%)	SPE (%)	AUC
SVM (Atlas-based)	DTI	85.87	86.88	84.82	0.8571	86.18	86.94	85.40	0.8585
H-FCN	DTI	86.75	86.70	86.80	0.8675	85.98	84.54	87.44	0.8606
nSEAL	DTI	87.46	84.17	88.46	0.8632	88.86	92.14	85.42	0.8878
DCNs	fMRI	90.54	89.65	91.46	0.9083	91.64	91.24	92.04	0.9192
N2EN	fMRI	93.45	92.27	94.67	0.9392	93.76	92.60	94.94	0.9396
SVM (Multi-kernel)	DTI & fMRI	95.60	94.20	97.05	0.9594	95.82	96.52	95.11	0.9596
HebrainGNN	DTI & fMRI	95.64	93.06	97.50	0.9528	95.97	95.83	96.28	0.9605
MME-GCN	DTI & fMRI	95.93	97.98	94.25	0.9612	95.88	95.83	96.33	0.9608
3D-CNN	DTI & fMRI	96.06	96.70	95.39	0.9592	96.03	95.84	96.22	0.9631
OLFG	DTI & fMRI	96.78	96.25	98.00	0.9712	96.73	96.08	97.77	0.9693
BID-MGE (without distances)	DTI & fMRI	95.91	97.43	92.84	0.9514	94.55	90.90	98.00	0.9445
BID-MGE	DTI & fMRI	99.07	98.47	99.97	0.9923	98.80	99.92	97.65	0.9897

The superior performance of our method compared with those multi-modal approaches may stem from the following facts. First, these multi-modal methods typically emphasize the internal relationships within brain networks, often overlooking the potential interactions between nodes across modalities. By contrast, our method can capture wider node interactions and preserve the characteristics unique to each modality through multilayer informativeness diffusion. Second, our method employs beta mapping to refine the vital connectivity of brain networks, which facilitates the extraction of more discriminative features during the diffusion process and plays a crucial role in improving classification performance. In summary, our results suggest that alterations in structural and functional connections are crucial for diagnosing neuropsychiatric disorders. Moreover, incorporating multi-modal brain networks significantly improves classification performance. It also implies that exploring wider node interactions between brain structures and functions and mining intrinsic characteristics of brain networks could further enhance the diagnosis of neuropsychiatric disorders.

### 3.6 Comparison with previous studies

In this section, we conducted a comparison with several available methods using neuroimaging data from the COBRE dataset (Mayer et al., 2013). The dataset includes structural magnetic resonance imaging (sMRI), fMRI, and DTI modalities. We collected 73 subjects for whom both DTI data and resting-state fMRI data are available, participants consist of 37 HC and 36 SZ. The ages of all subjects ranged from 20 to 65 years, and their age and gender distributions were not significantly different. Data acquisition parameters of DTI and fMRI can be found in Masoudi and Danishvar (2022). Data preprocessing is described above. The methods compared include single-modal methods and multi-modal methods. Table 3 reported the results of previous studies. Notably, the results of different methods are not directly comparable due to variations in the sample sizes, preprocessing methods, and data division. From Table 3, we observed the following points. First, multi-modal methods outperform single-modal methods due to the utilization of complementary information between modalities. Second, the performance of the BID-MGE method surpasses that of the existing method for most evaluation metrics. The enhancements attained by BID-MGE can be due to the incorporation of both complementary information and unique characteristics from various modalities. Third, beta mapping enhances the performance of our method, which further proves that beta mapping is effective in refining structural and functional connectivity information.

**Table 3:** Performance of our method and previous studies on the COBRE dataset (SZ vs. HC).

Study	Modality	Subject	ACC (%)	SEN (%)	SPE (%)
Huang et al. (2020a)	fMRI	67 HC, 53 SZ	82.4	91.30	72.50
Aggarwal et al. (2017)	fMRI	50 HC, 50 SZ	89	–	–
Chyzhyk et al. (2015)	fMRI	72 HC, 74 SZ	91.2	–	–
Silva et al. (2014)	sMRI and fMRI	75 HC, 69 SZ	94	–	–
Qureshi et al. (2017)	sMRI and fMRI	72 HC, 72 SZ	99.29	100.00	98.57
Masoudi and Danishvar (2022)	DTI and sMRI	81 HC, 64 SZ	99.50	99.75	97.13
BID-MGE (without beta mapping)	DTI and fMRI	37 HC, 36 SZ	97.60	95.36	99.60
BID-MGE (without distances)	DTI and fMRI	37 HC, 36 SZ	98.57	98.33	99.65
BID-MGE	DTI and fMRI	37 HC, 36 SZ	99.71	99.67	99.75

## 4 Discussion

### 4.1 Significance of results

The node representation proves to be a useful form for brain network analysis. Previous studies showed that neuropsychiatric disorders may result from abnormalities in some specific brain regions, thereby leading to alterations in structural and functional connectivity among brain regions (Klauser et al., 2017; Kim et al., 2019). To capture these changes, the BID-MGE method generates node representations with comprehensive information to characterize brain connectivity. BID-MGE exhibits three key differences compared with existing methods: (1) our method considers both complementary information and unique features from various modalities. (2) The traditional graph embedding methods are generally used for node classification and link prediction, rather than specifically for brain network analysis. Thus, these methods fail to take into account the integration of diverse neuroimaging modalities (e.g., SC and FC). (3) Our method incorporates beta mapping to refine SC and FC, effectively steering the diffusion process toward key brain regions that cause disease. The results in Tables 2, 3 illustrate that the proposed method enhances the classification performance. Additionally, our method also discovers several crucial brain regions associated with the disease, as depicted in Figure 4. For further details, Table 4 lists several brain regions exhibiting a value of *p* less than 0.05 after Bonferroni correction, consistent with previous research findings. The value of *p* is derived from a two-tailed *t*-test. Specifically, several brain regions have abnormalities in SZ and BD as displayed in Figures 4A,B, such as the middle frontal gyrus, orbital, cuneus, and paracentral lobule. This may be due to shared structural and functional dysfunctions in SZ and BD (Dong et al., 2017; Xia et al., 2019).

**Table 4:** The ROIs with significant differences (corrected value of *p* <0.05).

Group	Type	ROI	Full name	Related studies
SZ vs. HC	Structure	Precentral_R	Precentral gyrus	Zhou et al. (2005)
		Rectus_R	Gyrus rectus	Masaoka et al. (2020)
		Thalamus_L	Thalamus	Shimizu et al. (2008)
	Function	Precuneus_L	Precuneus	Hoptman et al. (2010)
		Supp_Motor_Area_L area	Supplementary motor area	Mashal et al. (2014)
BD vs. HC	Structure	Cuneus_L	Cuneus	Qiu et al. (2014)
		Frontal_Sup_Medial_L	Superior frontal gyrus, medial	Repple et al. (2017)
	Function	Paracentral_Lobule_R	Paracentral lobule	Zhang et al. (2020)
		Rolandic_Oper_R	Rolandic operculum	Lin et al. (2018)
		Lingual_L	Lingual gyrus	Zhong et al. (2016)
SZ vs. BD	Structure	Cingulum_Post_R	Posterior cingulate gyrus	Koo et al. (2008)
	Function	ParaHippocampal_L	Parahippocampal gyrus	Lui et al. (2015)
		Amygdala_L	Amygdala	Mahon et al. (2012)
		Bilateral Hippocampus	Hippocampus	Hall et al. (2010)

### 4.2 Prediction of clinical scores

**Figure 7:**
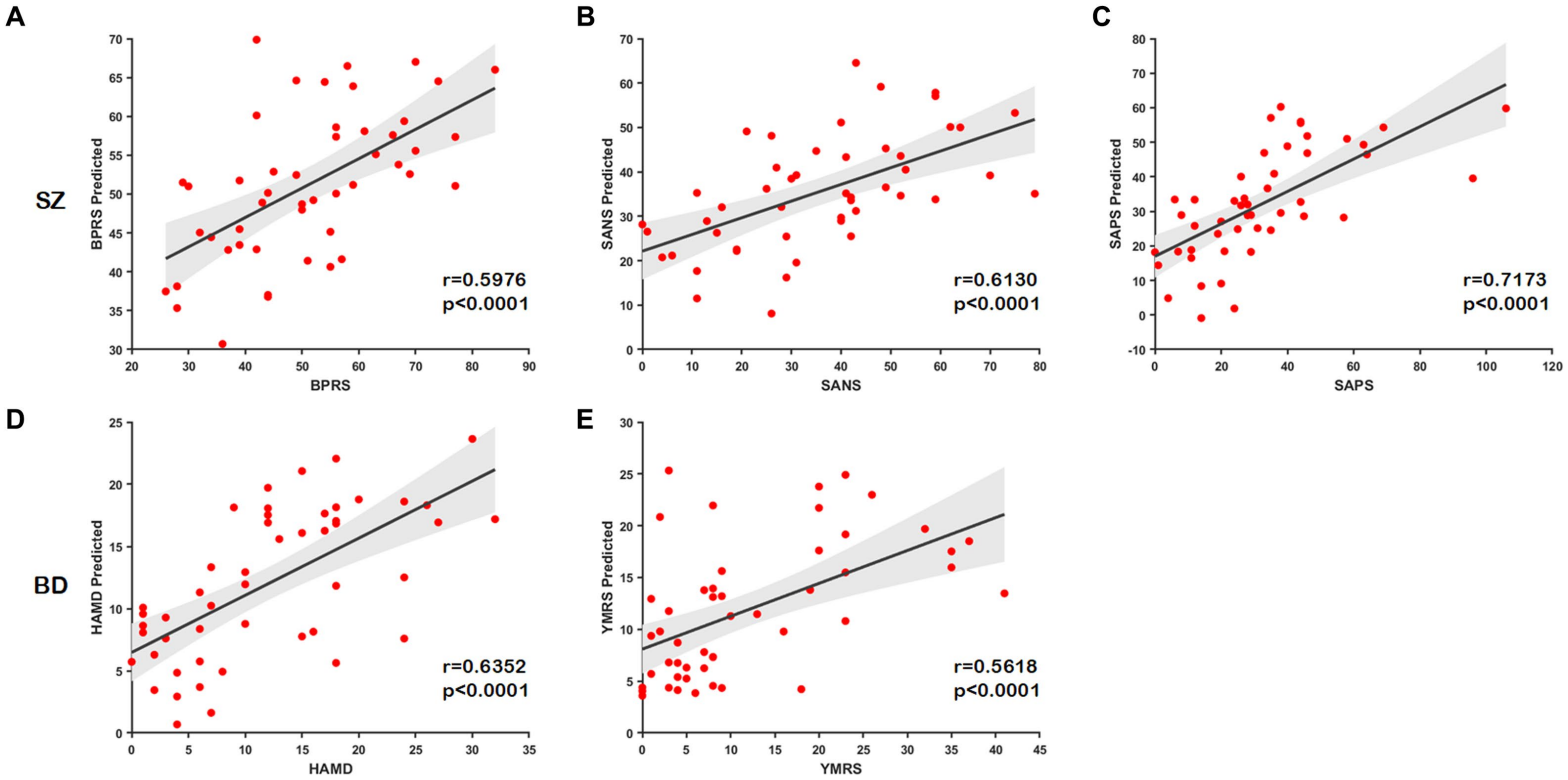
Scatter plots show correlations between the true scale scores and predictions. **(A–C)** The predicted scores of the scale of SZ. **(D,E)** The predicted scores of the scale of BD.

In this part, we examine the predictive ability of node distance for scale scores using connectome-based predictive modeling (CPM) (Shen et al., 2017). We concatenate the portions of node distance matrices with the same labels (e.g., $L^{s}$ , $L^{b}$ ) for three node-level templates to generate a new matrix as input to CPM. The correlation coefficient for retaining the number of nodes is *p* = 0.05. The predictive power of the node distance is estimated by the Spearman correlation between the predicted and true scale scores. All statistical tests are two-tailed. We found that node distances can effectively predict scale scores in unobserved subjects with SZ (BPRS, *r* = 0.5976, *p* < 0.0001; SANS, *r* = 0.6130, *p* < 0.0001; SAPS, *r* = 0.7173, *p* < 0.0001) and BD (HAMD, *r* = 0.6352, *p* < 0.0001; YMRS, *r* = 0.5618, *p* < 0.0001); the predicted and the true scale scores present a significant correlation as illustrated in Figures 7A–E. These results further indicate that our method effectively captures structural or functional brain alterations, and the node distance can act as an essential indicator to estimate the severity of the disease.

### 4.3 Time and space complexity of multilayer informativeness diffusion

For the time complexity of multilayer informativeness diffusion, the sampling process of the proposed method is the same as the standard random walk. During each iteration, sampling according to the transition probability, only one node sequence is generated per node. The sampling strategy uses alias sampling, which can complete one-step diffusion in O(1) time complexity (Grover and Leskovec, 2016), assuming that the count of iterations starting with every node and each truncated walk length is constant. Hence, the time complexity of completing the entire graph sampling is O(|V|) . For the space complexity of multilayer informativeness diffusion, the first is the space needed to store the multilayer brain network. As mentioned above, the edge number of the functional layer is θ times that of the structural layer (θ is a constant). Hence, our method needs O((θ+1)(|V|+|E|))=O(|V|+|E|) space to store the graph in the adjacency list format. In addition, alias sampling requires an additional O(|E|) space complexity. Thus, the total space complexity is O(|V|+2|E|) = O(|V|+|E|). The approximate time and space complexity of our method has no increase compared with classic random walk algorithms typically used for networks with single structural data.

### 4.4 Parameter sensitivity

The localized diffusion tends to capture higher-order proximity more effectively. Therefore, smaller values for p and larger values for q are typically favored for graph embedding within brain networks to learn superior node representation. In our experiments, we first fixed *p* and *q* at 0.1 and 1.6, respectively. Additionally, the two other parameters, *λ* and k , were set to 10 and d/2 , respectively. Then, we tested three main parameters of BID-MGE, including the functional layer network scale, distribution of beta mapping, and embedding dimension of BID-MGE. The network scale of the functional layer influences the computational time to process the multilayer brain network and the specificity of the learned node representations. The distribution of beta mapping determines its squeezing and expanding properties. The embedding dimension controls the integrity of reserving information.

#### 4.4.1 The functional layer network scale

**Figure 8:**
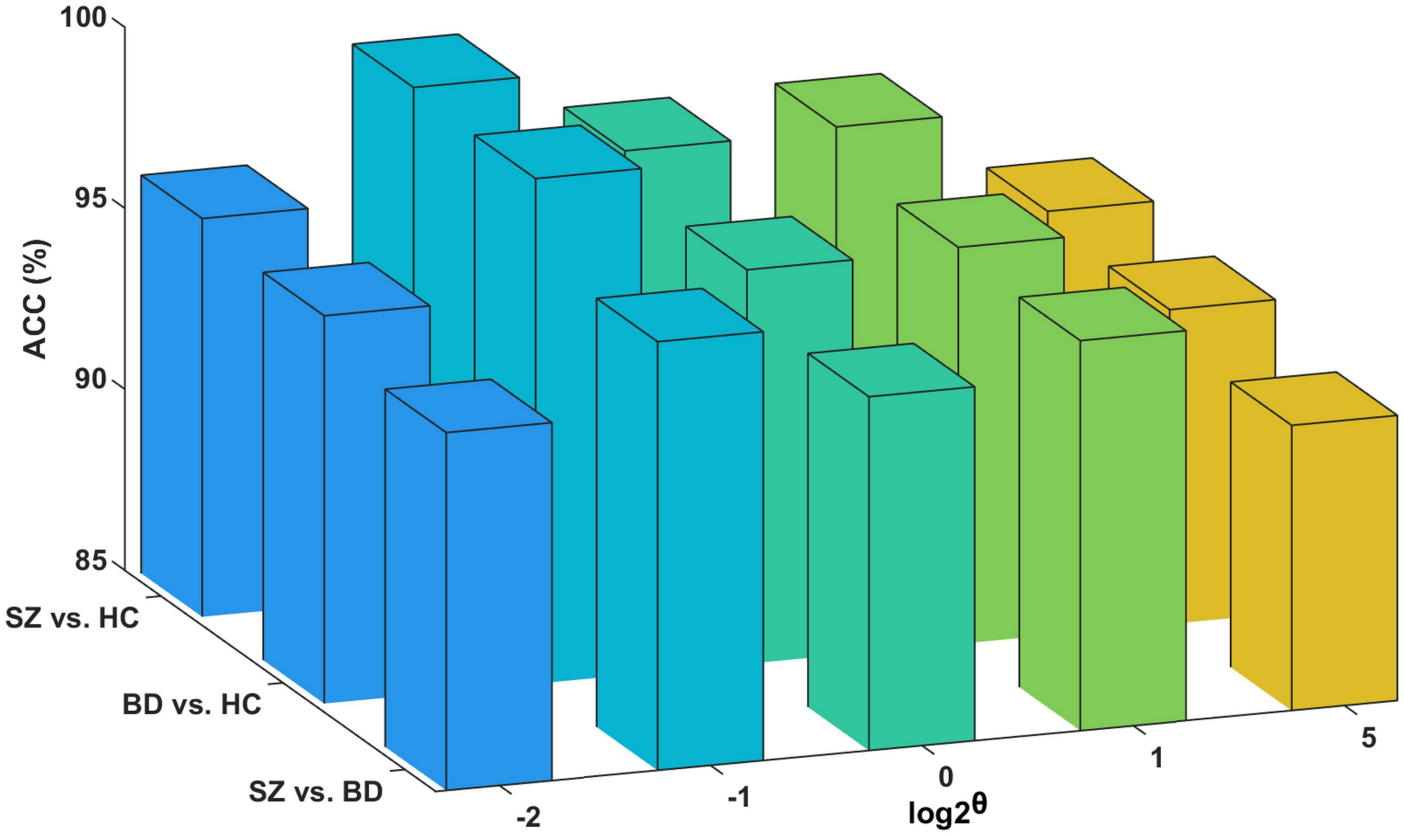
Influence of the functional layer network scale. Classification accuracies for the functional layer with different network scales.

To minimize the computation time in processing the multilayer brain network without compromising essential connectivity information, we use the structural layer as a benchmark to select the edges that form the functional layer. Figure 8 presents classification accuracies with different network scales of the functional layer. The best performance is obtained at *θ* = 0.5 for the three binary classifications (i.e., the functional layer is half the network scale of the structural layer). However, if the network scale of the functional layer is as small as *θ* = 0.25, it may lead to an incomplete aggregation of the semantic neighborhood information of the nodes. Consequently, we set θ = 0.5 as the optimal parameter of the network scale.

#### 4.4.2 The distribution of beta mapping

**Figure 9:**
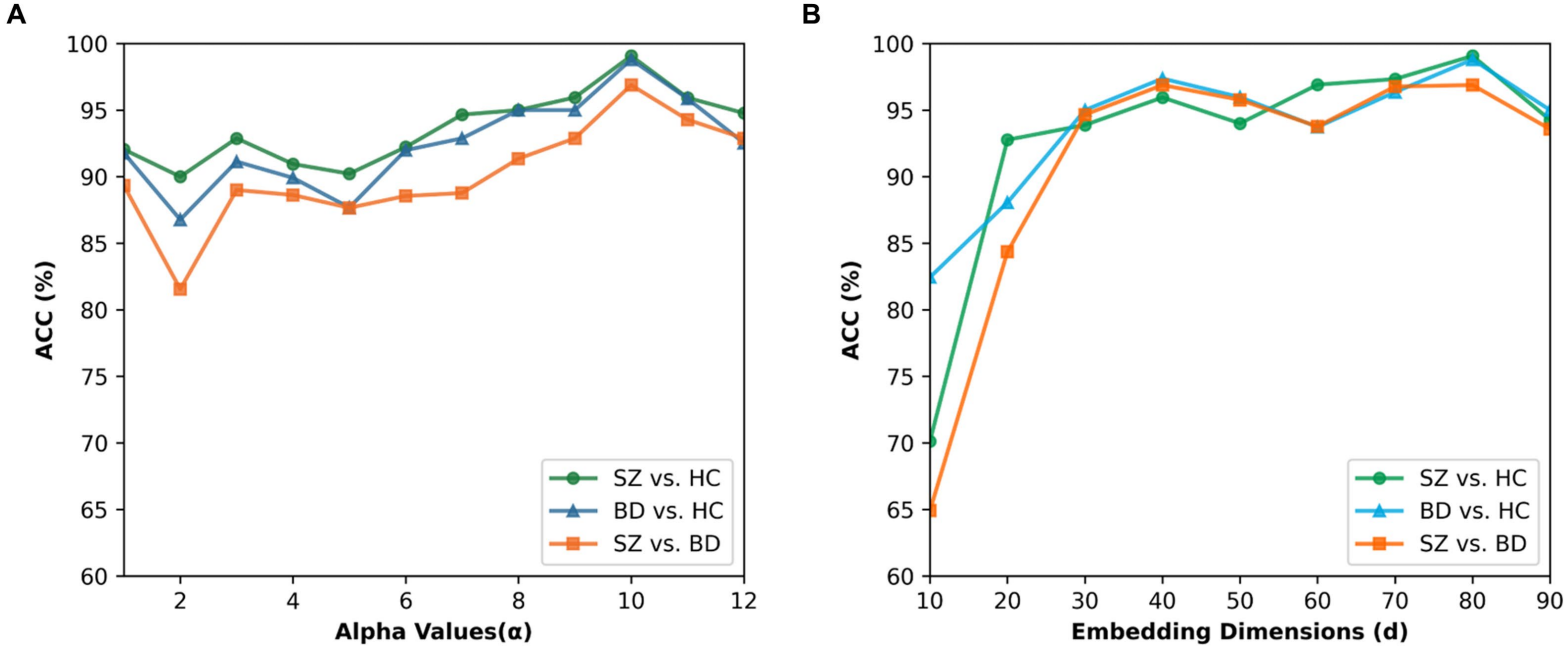
Effect of the parameter alpha and embedding dimension. **(A)** Classification accuracies for different alpha values of beta mapping. **(B)** Classification accuracies for different embedding dimensions.

In beta mapping, the parameters α and β are used to control the shape of the distribution, thereby altering its compression and expansion properties. We want to strengthen the connections that matter and weaken the ones that do not. In addition, for α ≥ 1 and β < 1, the value of Beta tends to move toward infinity as x is close to 1 and so does ψ(x), thereby causing irrational connections existing in the brain network. Therefore, we only consider the case in which the beta mapping monotonically grows with an upper bound (i.e., α>1 and β=1 ). Figure 9A presents the results for α values ranging from 1 to 12 and β values of 1 in all cases. The best performance for the three binary classifications is achieved at *α* = 10. When *α* > 10, the classification accuracies are gradually decreased. In our study, 10 is finally chosen as the value of parameter α.

#### 4.4.3 The embedding dimension of node representation

To explore the impact of the embedding dimension on the proposed method, we tested the BID-MGE method with different embedding dimensions and the results are depicted in Figure 9B. We noticed that optimal performance occurs at *d* = 80 for all classifications. Beyond this dimension, the accuracies decline due to the involvement of redundant or interfering features.

### 4.5 The effectiveness of beta mapping

**Figure 10:**
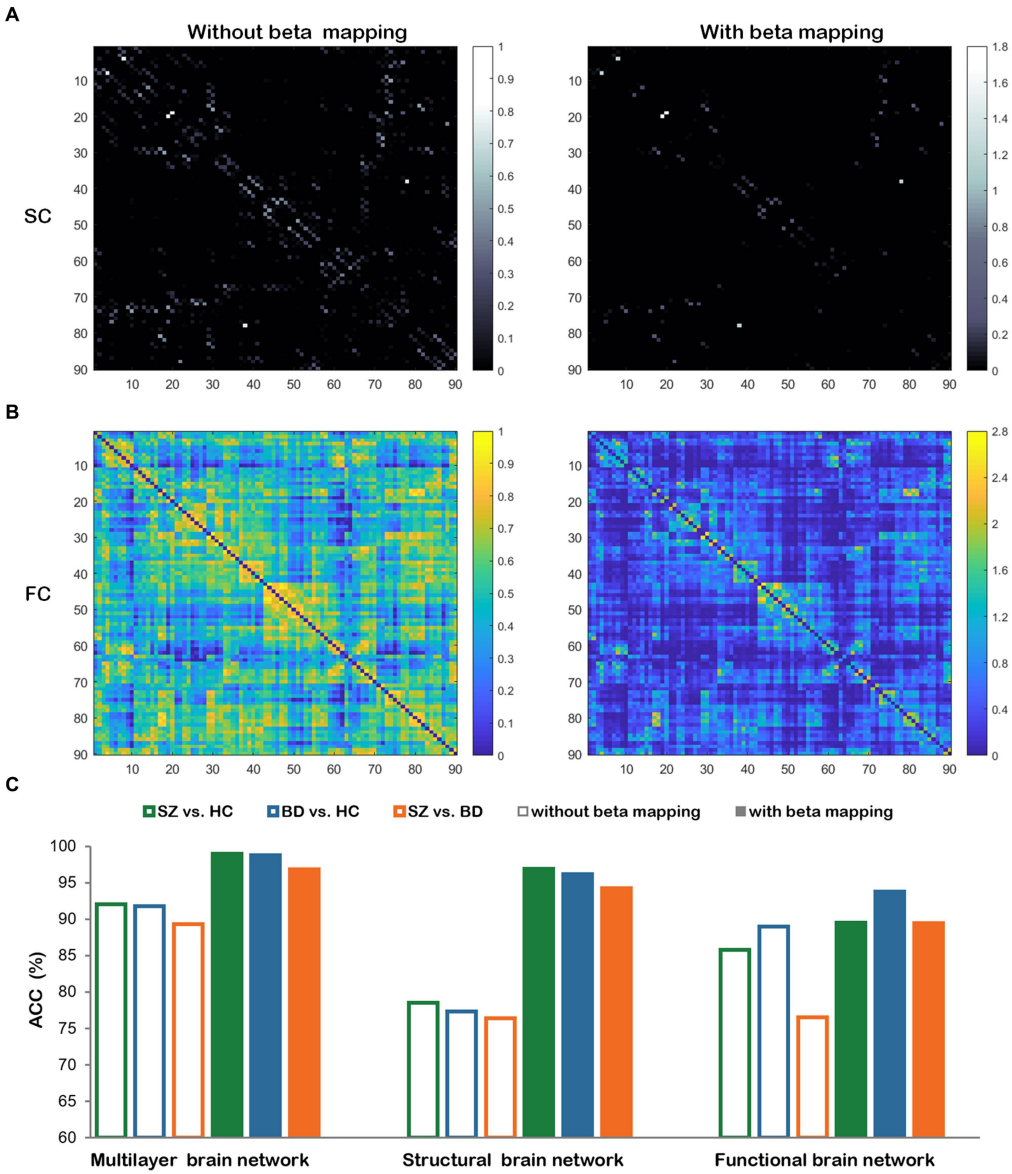
The comparisons with and without beta mapping. **(A)** Structural connectivity matrix. **(B)** Functional connectivity matrix. **(C)** Classification accuracies for three classification tasks with and without beta mapping in three brain networks.

The beta mapping’s squeezing and expanding properties make it possible to increase critical connectivity and weaken negligible information. In Figures 10A,B, the SC and FC of a healthy subject are illustrated. These images display the changes with and without beta mapping. We observed that the number of strength connections decreased, which promotes the diffusion process to focus more on key brain regions. From Figure 10C, we can find that the classification accuracies are remarkably improved after employing beta mapping; the results indicate that beta mapping contributes to the identification of diseases. Specifically, beta mapping significantly improves the accuracy of classification by structural brain networks. The reason is the small differences in the connection strengths of the original structural connectivity. After applying beta mapping, these differences are amplified and some interfering information is removed, allowing more discriminative features to be extracted in the diffusion process.

### 4.6 Limitations and future work

There are three primary limitations in the current study. First, brain regions are defined using only the AAL template. In future studies, we will validate the efficacy of the proposed method using other brain region templates, such as the Human Brainnetome Atlas (Fan et al., 2016). Second, our method only considers connectivity information among brain regions even though brain regions still have some attributes, such as cortical thickness, anisotropy index, ReHo, and ALFF, which are also crucial for diagnosing neuropsychiatric disorders. Therefore, we will combine brain attributes and brain connectivity to further improve neuropsychiatric disorder diagnosis. Third, BD episodes include different phases (e.g., manic, depressive, or mixed). In our study, we do not consider the different phases of BD. Different phases may have different brain activities, necessitating further studies in the future.

## 5 Conclusion

In this study, we propose a novel brain network analysis method based on multiple modalities, which integrates SC and FC by intelligently traversing the nodes between structural and functional layers in a diffusion manner. Our approach takes full advantage of the complementary information and unique characteristics provided by various modalities and generates node representations with holistic information. Moreover, beta mapping allows the refined connectivity to encompass more valuable information, which further guides the diffusion process to concentrate on crucial brain regions to learn discriminative features. Experimental results on neuropsychiatric disorders validate the efficacy of our method.

## Data availability statement

The datasets presented in this study can be found in online repositories. The names of the repository/repositories and accession number(s) can be found in the article/Supplementary material.

## Ethics statement

The studies involving humans were approved by the Poldrack Lab and Center for Reproducible Neuroscience at Stanford University. The studies were conducted in accordance with the local legislation and institutional requirements. The participants provided their written informed consent to participate in this study.

## Author contributions

YH: Methodology, Writing – original draft. YL: Formal analysis, Writing – review & editing. YY: Data curation, Writing – original draft. XZ: Data curation, Writing – original draft. WY: Data curation, Writing – original draft. TL: Validation, Writing – review & editing. YN: Formal analysis, Writing – review & editing. TinY: Validation, Writing – review & editing. XL: Formal analysis, Writing – review & editing. DL: Supervision, Writing – review & editing. JX: Supervision, Writing – review & editing. BW: Conceptualization, Writing – review & editing. TiaY: Project administration, Writing – review & editing. MX: Investigation, Writing – review & editing.
