# Supplementary material for: Beta-informativeness-diffusion multilayer graph embedding for brain network analysis
Source: Front Neurosci. 2024 Mar 8;18:1303741. doi: 10.3389/fnins.2024.1303741 (PMC10957763; doi:10.3389/fnins.2024.1303741)
Supplement: Supplementary file 1 [file Table_1.DOCX]

| Distances | SZ vs. HC | | | |  | BD vs. HC | | | |
| --- | --- | --- | --- | --- | --- | --- | --- | --- | --- |
|  | ACC (%) | SEN (%) | SPE (%) | AUC |  | ACC (%) | SEN (%) | SPE (%) | AUC |
| DTW | 64.55 | 6866 | 63.03 | 0.6584 |  | 63.37 | 65.20 | 62.36 | 0.6378 |
| Chebyshev | 69.33 | 7316 | 68.95 | 0.7105 |  | 66.88 | 61.35 | 70.23 | 0.6579 |
| Euclidean | 95.77 | 9425 | 97.89 | 0.9607 |  | 93.31 | 95.42 | 91.77 | 0.9360 |
| Manhattan | 97.00 | 96.66 | 98.00 | 0.9733 |  | 96.88 | 96.66 | 97.50 | 0.9708 |
| Cosine (our method) * | **99.07** | **98.47** | **99.97** | **0.9923** |  | **98.80** | **99.92** | **97.65** | **0.9897** |

**TABLE S1 |** Performance comparison of different distance calculation methods

DTW (Dynamic Time Warping) Distance is a method used to measure the similarity between two time series, which has a relatively high time complexity. DTW is mainly used to deal with the situation where two time series are slightly offset on the time axis, and it is often used in speech recognition, handwriting recognition, and bioinformatics. Poor performance on clustering and classification problems in machine learning.

Chebyshev Distance is the maximum absolute value of the coordinate difference in all dimensions. It represents the maximum difference between two points on all coordinate axes. The characteristic of Chebyshev Distance is to consider the maximum difference on all dimensions, which also causes the loss of some subtle feature information, and is commonly used in fields such as image processing.

Euclidean Distance is the straight-line distance between two points in Euclidean space, often used to measure the similarity or difference between data points. In high dimensional space, Euclidean Distance is affected by the so-called "dimensional disaster".

Manhattan Distance is the sum of the absolute values of the coordinate difference in each dimension, and the characteristic of Manhattan Distance is to consider only the difference on the coordinate axis, not the straight path. Unlike Euclidean Distance, Manhattan Distance is not affected by "dimensional disaster" in high dimensional space.

Cosine Distance is a measure of the similarity of the angle between two vectors. It measures the difference in direction between two vectors, regardless of their length. In machine learning, Cosine Distance is also commonly used for clustering and classification problems. Since Cosine Distance takes into account only the direction and not the length, it is particularly useful for similarity measures of vectors in high-dimensional Spaces.

The results are shown in Table S1, because our method concatenates the features of all nodes together as the network-level feature, resulting in its relatively large dimension. Hence, better results are achieved by applying Manhattan Distance and Cosine Distance metrics, while this experiment is mainly used for the classification task, so Cosine Distance is more appropriate in our experiment.
